# H_2_O_2_ production on a carbon cathode loaded with a nickel carbonate catalyst and on an oxide photoanode without an external bias†

**DOI:** 10.1039/d1ra01045j

**Published:** 2021-03-17

**Authors:** Soichi Takasugi, Yugo Miseki, Yoshinari Konishi, Kotaro Sasaki, Etsuko Fujita, Kazuhiro Sayama

**Affiliations:** Global Zero Emission Research Center (GZR), National Institute of Advanced Industrial Science and Technology (AIST) Central 5, 1-1-1 Higashi Tsukuba Ibaraki 305-8565 Japan k.sayama@aist.go.jp; Chemistry Division, Brookhaven National Laboratory Upton New York 11973-5000 USA

## Abstract

Efficient H_2_O_2_ production both on a carbon cathode modified with various metal salts and on an oxide photoanode was investigated. The cathodic current density and faradaic efficiency for H_2_O_2_ production (FE(H_2_O_2_)) on a carbon cathode in KHCO_3_ aqueous solution were significantly improved by the loading of an insoluble nickel carbonate basic hydrate catalyst. This electrode was prepared by a precipitation method of nickel nitrate and KHCO_3_ aqueous solution at ambient temperature. The nickel carbonate basic hydrate electrode was very stable, and the accumulated concentration of H_2_O_2_ was reached at 1.0 wt% at a passed charge of 2500C (the average FE(H_2_O_2_) was 80%). A simple photoelectrochemical system for H_2_O_2_ production from both the cathode and a BiVO_4_/WO_3_ photoanode was demonstrated without an external bias or an ion-exchange membrane in a one-compartment reactor under simulated solar light. The apparent FE(H_2_O_2_) from both electrodes was calculated to be 168% in total, and the production rate of H_2_O_2_ was approximately 0.92 μmol min^−1^ cm^−2^. The solar-to-chemical energy conversion efficiency for H_2_O_2_ production (STC_H_2_O_2__) without an external bias was approximately 1.75%.

## Abstract 2

Efficient H_2_O_2_ production both on a nickel carbonate cathode and a photoanode using only H_2_O, O_2_, and solar light, was achieved without using an external bias or a membrane.

## Graphical abstract

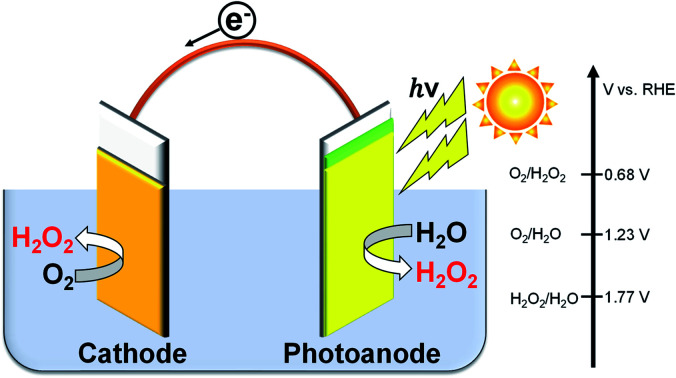

## Introduction

Hydrogen peroxide (H_2_O_2_) is an environmentally friendly and important chemical oxidant that has been widely applied to pulp bleaching, waste treatment, chemical synthesis of peroxides, and food sterilization.^1–8^ The anthraquinone redox process using H_2_ and O_2_ gases has been used for the industrial large-scale production of H_2_O_2_.^2,9^ However, there are many problems in this process: a large amount of energy requirement and H_2_ consumption in the complicated system producing a huge amount of CO_2_ emission, the usage of harmful organic solvents, the requirement of concentration, and transformation from the central process to consumption area. Therefore, some distributed and small-scale electrochemical H_2_O_2_ production processes in aqueous solution have been widely investigated.^5,10–22^ The on-site electrochemical process has many advantages, such as safety without H_2_ usage, no organic solvent separation, and controllability on the production amount and concentration for demand. Two kinds of reactions for H_2_O_2_ production are present in the electrochemical process: reductive H_2_O_2_ production from O_2_ on a cathode (eqn (1)), and oxidative H_2_O_2_ production from H_2_O on an anode (eqn (2)).

**Fig. 1:**
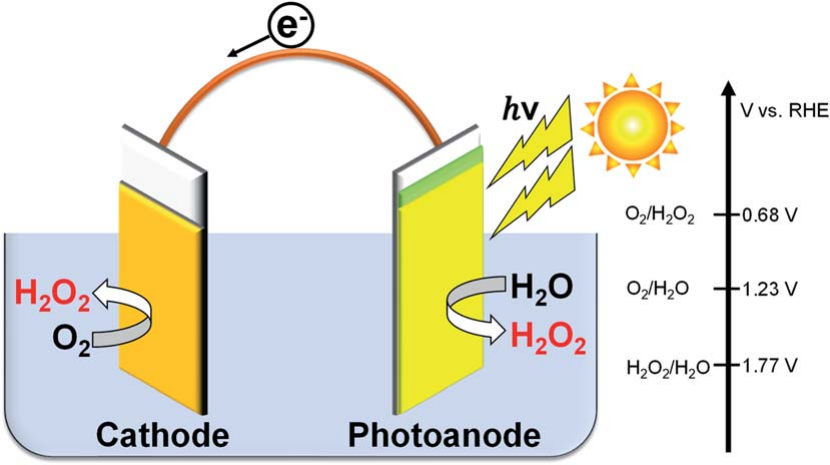
H_2_O_2_ production on both electrode sides in a one-compartment cell under illumination without an external bias or an ion-exchange membrane.

There are many reports on the reductive H_2_O_2_ production from O_2_.^23–30^ In contrast, the oxidative H_2_O_2_ production and accumulation from H_2_O are very difficult. However, we and others have reported that H_2_O_2_ can be accumulated when KHCO_3_ aqueous solution is used in electrochemical and photoelectrochemical processes.^19–22,31–35^ It is advantageous and highly efficient to produce H_2_O_2_ on both electrode sides by combining the cathodic and anodic reactions, as shown in eqn (3), compared to the production on each electrode side under the same electric charge. The apparent faradaic efficiency for the H_2_O_2_ production (FE(H_2_O_2_)) could reach 200% in total (100% + 100%) if the production occurred on both sides of the electrode.^36^ We have previously demonstrated that the H_2_O_2_ production can take place on a BiVO_4_/WO_3_ photoanode and an Au cathode at near-neutral pH in KHCO_3_ aqueous solution using a simple one-compartment cell without an external bias, as shown in Fig. 1.^19^ The apparent faradaic efficiency was 140% in total (FE(H_2_O_2_) = 90% and 50% on the Au cathode and the BiVO_4_/WO_3_ photoanode, respectively). The band gap of BiVO_4_ is 2.4 eV, and the theoretical maximum photocurrent is reported to be 7.5 mA cm^−2^.^37^ Unfortunately, the current density of the Au cathode, a novel metal electrode, was low (−0.18 mA cm^−2^ at +0.5 V (*vs.* RHE)). Therefore, improved systems are needed for practical applications.

Cathode reaction:

1 O_2_ + 2H^+^ + 2e^−^ → H_2_O_2_, *E*(O_2_/H_2_O_2_) = +0.68 V (*vs.* RHE)

Anode reaction:

2 2H_2_O → H_2_O_2_ + 2H^+^ + 2e^−^, *E*(H_2_O_2_/H_2_O) = +1.77 V (*vs.* RHE)

Eqn (1) + (2)

3 2H_2_O + O_2_ → 2H_2_O_2_ two-electron process

As for noble-metal-free cathodes, there are several reports on the use of carbon-based materials modified with inexpensive metal compounds mainly at basic pH.^38–41^ However, efficiencies for the H_2_O_2_ production with these cathodes were low at near-neutral pH, and an ion-exchange membrane was essential when the pH values of the anodic and cathodic electrolyte solutions were different.^21,22^ Recently, we reported that a carbon cathode modified with a biomass-derived W-based electrocatalyst exhibited a relatively high cathodic current for the reductive H_2_O_2_ production in a near-neutral KHCO_3_ aqueous solution in a two-compartment cell, but the FE(H_2_O_2_) and the partial current density for H_2_O_2_ production were not enough.^20^

In this paper, we pursued developing more efficient H_2_O_2_ production systems on a photoanode and a noble-metal-free cathode in a KHCO_3_ aqueous solution without an ion-exchange membrane or an external bias in a one-compartment cell. The system using a single electrolyte in a one-compartment cell without any membrane is very simple. It was found that nickel carbonate on a carbon-based cathode showed excellent electrocatalytic activity on both FE(H_2_O_2_) and the partial current density for H_2_O_2_ production in a KHCO_3_ solution. A water-insoluble nickel carbonate electrocatalyst was easily prepared *in situ* from a nickel salt and KHCO_3_ aqueous solution at ambient and mild conditions. The apparent solar-to-chemical energy conversion efficiency for H_2_O_2_ production (STC_H_2_O_2__) on a combination system of the BiVO_4_/WO_3_ photoanode and the cathode loaded with nickel carbonate electrocatalyst without external bias reached 1.75%, which is the highest among all reported values so far.

## Experimental section

### Fabrication of the carbon cathode loaded with various metals

A water-repellent-treated carbon paper (abbreviated as CP; 1.0 cm^2^, TORAY, TGP-H-090) was used as the substrate plate of the cathode. A conductive carbon powder of Ketjenblack EC600JD (abbreviated as KB; Lion Specialty Chemicals Co., Ltd.)^42–44^ was used with various electrocatalysts on CP. KB powder loaded with various metal salts as an electrocatalyst (abbreviated as Met/KB) was prepared by an impregnation method. The metal salts used were Cr(ii) nitrate nonahydrate, Mn(ii) nitrate tetrahydrate, Fe(iii) nitrate nonahydrate, Co(ii) nitrate hexahydrate, Ni(ii) nitrate hexahydrate, Cu(ii) nitrate trihydrate, Zn(ii) nitrate hexahydrate, Ga(iii) nitrate *n*-hydrate, hexaammonium heptamolybdate tetrahydrate, and ammonium tungstate *para*-pentahydrate (purchased from Fujifilm Wako Pure Chemicals Corporation).

A mixture of 100 mg of KB powder and 0–1000 mg of various metal salts was added to 20 mL of H_2_O. The suspension was thoroughly dispersed by ultrasonication for 30 min and dried overnight in a heating oven at 353 K under an ambient pressure. The ratio of a loaded metal salt of *x* wt% to the weight of the KB powder was abbreviated as Met_*x*_/KB. For example, a loaded metal salt of 10 and 100 wt% *vs.* the weight of the KB powder were abbreviated as Met_10_/KB and Met_100_/KB, respectively. A mixture of 1.5 mg (standard amount) of the electrocatalyst (KB, Met/KB) and 0.75 mg of 20 wt% Nafion solution (Sigma-Aldrich Co., USA) was dispersed in 0.5 mL of ethanol, and loaded on one side of the CP substrate (area 1.0 cm^2^) at room temperature. This cathode loaded with an electrocatalyst and KB powder on CP was dried at 333 K for 30 min in a heating oven.

### HNO_3_ treatment for KB powder

HNO_3_-treated KB powder (abbreviated as KB_HNO3_) was prepared by an immersion process. To 38.3 g of concentrated HNO_3_ aqueous solution (60%, Fujifilm Wako Pure Chemical Corporation), 1000 mg of KB powder was added. The suspension was thoroughly heated at 353 K for 24 h with a string at 300 rpm in a heating oven. They were collected by suction filtration and thoroughly washed with distilled water. Metal-salt loading on KB_HNO3_ (abbreviated as Met/KB_HNO3_) was prepared and coated on CP as the cathode by the same process.

### Fabrication of the BiVO_4_/WO_3_ photoelectrode

The BiVO_4_/WO_3_ photoelectrode used in this study was synthesized using the method previously reported by Fuku,^45^ and was prepared on a F-doped SnO_2_ (FTO) glass substrate by spin-coating method. The precursor of the WO_3_ layer was loaded by spin coating (1000 pm, 15 s, 200 μL per 12 cm^2^) on FTO, and then calcined at 773 K for 30 min. Spin coating of the WO_3_ layer was repeated twice using *N*,*N*-dimethylformamide (DMF) solutions of tungsten hexachloride (WCl_6_, 4 N; Kojundo Chemical Laboratory, Co., Ltd.) adjusted to 504 and 252 mM for the first and second coat, respectively. The BiVO_4_ layer was also fabricated by spin coating (500 rpm, 15 s, 400 μL per 12 cm^2^) on the WO_3_ layer, and calcined at 773 K for 30 min. The spin coating of the BiVO_4_ layer was repeated three times using 0.4 or 0.6 M BiVO_4_ precursor solution. The first layer of BiVO_4_ used 0.4 M BiVO_4_ precursor solution, and the rest of the layer used 0.6 M solution. The BiVO_4_ precursor solution, adjusted to 100 mM, was prepared by dissolving bismuth oxide (BiO_1.5_) solution (Symmetrix Co., USA) and a vanadium oxide (VO_2.5_) solution (Symmetrix Co., USA) in butyl acetate with a Bi/V molar ratio of 1.0. To the BiVO_4_ solution, 10 wt% butyl acetate solution of ethyl cellulose was added as a thickening agent with a (BiVO_4_ solution)/(ethyl cellulose solution) volume ratio of 1.5.

## Electrochemical measurements and quantification method

### Electrochemical measurement on the cathode

The electrochemical properties of the electrocatalyst cathode were evaluated in a 2.0 M KHCO_3_ aqueous solution (pH 8.8) with O_2_ bubbling (50 mL min^−1^) with an ice bath (273–278 K) by using an electrochemical analyzer (Hokuto Denko, HZ-7000). The current–potential (*I*–*E*) characteristics were measured *via* a three-electrode method using a two-compartment cell. The scan rate was set at 2 mV s^−1^. The volume of the electrolyte solutions of the anode and cathode chambers was 35 mL with stirring. The reference electrode used was 3 M Ag/AgCl, and the counter electrode was a Pt wire. Between the anode and cathode chambers, Aciplex (Asahi KASEI) was used as an ion-exchange resin. After the reaction was completed, 1.1 mL of the reaction solution was taken out using a syringe. The schematic illustration of a two-compartment cell is shown in Fig. S1(a).† All potentials in this paper were quoted with respect to the reversible hydrogen electrode (RHE), according to the Nernst equation (eqn (4)).

4 *E*(*vs.* RHE) = *E*(*vs.* Ag/AgCl) + 0.0591 × pH + 0.197

An accumulation experiment for H_2_O_2_ was conducted at a constant applied bias of +0.5 V *vs.* RHE.

### Simultaneous H_2_O_2_ production on both the photoanode and cathode without an external bias or membrane

The simultaneous production of H_2_O_2_ from H_2_O oxidation at the photoanode and O_2_ reduction at the cathode was performed without applying any external bias using a two-electrode system composed of a BiVO_4_/WO_3_ photoanode (irradiation area of 0.2 cm^2^ with a white board behind the photoanode) and a Ni_10_/KB_HNO_3__ cathode (catalyst loading 1.5 mg cm^−2^, area 0.2 cm^2^). An aqueous solution of KHCO_3_ (2.0 M) was used as an electrolyte (200 mL), and CO_2_ and O_2_ gases were each bubbled for 50 mL min^−1^ into the one-component cell with a pH value of 8.0 in an ice bath (273–278 K). An ice bath was used under CO_2_ bubbling to obtain the optimum reaction conditions for the stability of the photoanode. It was confirmed that O_2_ gas diffusion is not the rate-limiting factor under our conditions of <10 mA cm^−2^. A solar simulator calibrated to AM 1.5G (1 SUN, 0.1 W cm^−2^) was used as the light source. The schematic illustration of our reaction system in a one-compartment cell without membrane is shown in Fig. S1(b).†

### Characterization and quantification method

The prepared electrodes were characterized by X-ray fluorescence (XRF, Rigaku, Super mini200) measured in a vacuum with a wavelength dispersive spectrometer with Pd–K radiation. The crystal structures of the samples were investigated by X-ray diffraction (XRD, Malvern Panalytical, Empyrean) using Cu Kα radiation at 40 kV and 40 mA. Transmission electron microscopy and energy dispersive X-ray spectroscopy (TEM and EDS, Philips, Tecnai Osiris) measurements were carried out with a field emission gun operating at 200 kV.

The amount of produced H_2_O_2_ was quantified *via* UV-visible spectroscopy (TECAN, Infinite 200 PRO). Then, 1.0 mL of sample was added to 0.9 mL of 3.0 M HCl aqueous solution and 0.1 mL of FeCl_2_ in 1.0 M HCl aqueous solution and quantified from Fe^3+^ colorimetry (*λ* = 330 nm), as we reported previously.^19,20,32,44^ The faradaic efficiency for H_2_O_2_ production (FE(H_2_O_2_)) was calculated using eqn (5):

5 FE(H_2_O_2_)% = [amount of produced H_2_O_2_] × 100 × 2/[amount of passed electrons]

We confirmed that the error range of the FE(H_2_O_2_) value was around ±2%.

The apparent partial current density for H_2_O_2_ production at a constant potential (*J*_ap_(H_2_O_2_)) was calculated using eqn (6):

6 *J*_ap_(H_2_O_2_) = *J*(Total) × FE(H_2_O_2_)

Here, *J*(Total) is the total current density at a constant potential and FE(H_2_O_2_) is the average faradaic efficiency at the passed charge of a constant potential. *J*_ap_(H_2_O_2_) represented the actual H_2_O_2_ production rate.

The turnover number was calculated using eqn (7). The amount of nickel was measured by XRF.

7 Turnover number = [amount of produced H_2_O_2_ (in moles)]/[amount of nickel (in moles)]

The apparent solar-to-chemical energy conversion efficiency (STC) for the H_2_O_2_ and O_2_ production on the BiVO_4_/WO_3_ photoanode (irradiation area of 0.2 cm^2^ with a white board behind the photoanode) and on the Ni_10_/KB_HNO_3__ cathode (catalyst loading 1.5 mg cm^−2^, area 0.2 cm^2^) was calculated using eqn (8):^35^

8 STC_H_2_O_2__(%) = [(*V*_H_2_O_2__ × Δ*G*) × 100]/Int

*V*_H_2_O_2__ is the H_2_O_2_ production rate (in moles per second per square centimeter), Δ*G* is the Gibbs free energy from eqn (3) (Δ*G* = +116.8 kJ mol^−1^),^46^ and Int is the intensity of the incident simulated solar light (0.1 W s^−1^ cm^−2^).

## Results and discussion

Table 1 shows the *J*(Total) and the FE(H_2_O_2_) through a O_2_ reduction reaction on the KB/CP substrate cathodes modified with various metal salts (Met_100_/KB and Met_10_/KB, 100 and 10 wt% of metal salt in KB powder) in a 2.0 M KHCO_3_ aqueous solution under O_2_ bubbling condition. Here, FE(H_2_O_2_) and *J*_ap_(H_2_O_2_) were evaluated at the passed charge after 5C at +0.5 V. The *J*(Total) of the CP substrate was negligibly small. On the other hand, the *J*(Total) largely increased when KB powder was loaded on the CP substrate. KB powder has a high surface area (1270 m^2^ g^−1^) and high conductivity.^47^ It was surmised that the electron could be transferred through the conductive KB powder network, and that the reduction for H_2_O_2_ production could take place on the KB surface. The optimum loading amount of KB powder on the CP substrate was around 1.5 mg cm^−2^, and the current did not increase by further loading (Fig. S2†). The exfoliation of the powder from the CP substrate was observed by overloading (Fig. S2d†). Therefore, the loading amount of Met_10_/KB and Met_100_/KB on the CP substrate was fixed at 1.5 mg cm^−2^ in all experiments. The values of *J*(Total), FE(H_2_O_2_) and *J*_ap_(H_2_O_2_) were changed by the modification of various metal salts on the KB cathode. FE(H_2_O_2_) decreased by modification of Fe, Cu, Cr, Mn, Co, Ga, or W, compared to pristine KB powder and increased by modification with Mo_100_/KB, Ni_100_/KB, Ni_10_/KB, Zn_100_/KB, and Zn_10_/KB. As for the *J*(Total), most of the total current was improved by modification of these metal salts. The two-electron reduction for the H_2_O_2_ production reaction from O_2_ (eqn (1)) was competitive with other undesirable reactions: four-electron reduction of O_2_ to H_2_O (eqn (9)) and successive reduction of H_2_O_2_ to H_2_O (eqn (10)).

9 O_2_ + 4H^+^ + 4e^−^ → 2H_2_O, *E*(O_2_/H_2_O) = +1.23 V (*vs.* RHE)

10 H_2_O_2_ + 2H^+^ + 2e^−^ → 2H_2_O, *E*(H_2_O_2_/H_2_O) = +1.77 V (*vs.* RHE)

**Table 1:** FE(H_2_O_2_), *J*(Total), and *J*_ap_(H_2_O_2_) of Met_100_/KB and Met_10_/KB cathodes

Met/KB^a^	FE(H_2_O_2_)^b^/%	*J*(Total)^c^/mA cm^−2^	*J*_ap_(H_2_O_2_)/mA cm^−2^
CP substrate only	—	5.4 × 10^−4^	—
KB	59	−4.0	−2.4
Fe_100_/KB	4	−11.5	−0.4
Fe_10_/KB	13	−10.5	−1.4
Cu_100_/KB	9	−8.5	−0.8
Cu_10_/KB	42	−8.5	−3.6
Cr_100_/KB	32	−5.1	−1.6
Cr_10_/KB	43	−6.2	−2.9
Mn_100_/KB	1	−4.2	−0.04
Mn_10_/KB	3	−5.2	−0.2
Co_100_/KB	20	−3.3	−0.7
Co_10_/KB	15	−8.9	−1.3
Ga_100_/KB	54	−3.5	−1.9
Ga_10_/KB	56	−5.4	−3.0
W_100_/KB	33	−4.6	−1.5
W_10_/KB	44	−6.9	−3.0
Mo_100_/KB	36	−3.7	−1.3
Mo_10_/KB	67	−5.0	−3.4
Ni_100_/KB	66	−5.3	−3.5
Ni_10_/KB	85	−8.1	−6.9
Zn_100_/KB	71	−4.4	−3.1
Zn_10_/KB	65	−6.9	−4.5

a Met_100_/KB or Met_10_/KB: various metal salts loaded on KB powder (100 or 10 wt% of the loading amount of precursor metal salt (Met) *vs.* KB powder).

b The FE(H_2_O_2_) was measured at the passed charge after 5C.

c The *J*(Total) was measured at a constant potential of +0.5 V (*vs.* RHE).

The theoretical reduction potentials of the four-electron reduction of O_2_ (+1.23 V, eqn (9)) and successive reduction of H_2_O_2_ (+1.77 V, eqn (10)) were significantly positive compared to that of H_2_O_2_ production *via* two-electron reduction of O_2_ (+0.68 V, eqn (1)). In the case of the *J*(Total) improvement with decreasing FE(H_2_O_2_), the *J*_ap_(H_2_O_2_) was not significantly improved. It was surmised that these undesirable reactions might be accelerated by the modification of these metal nitrates or ammonium salt of Cu, Cr, Mn, Co, Ga, W, and Mo. On the other hand, the *J*(Total) and FE(H_2_O_2_) were both increased when Ni or Zn nitrate was modified. In particular, the Ni_10_/KB cathode showed the highest performance of *J*_ap_(H_2_O_2_) on the H_2_O_2_ production (−6.9 mA cm^−2^) among all metal salts in Table 1. The *J*_ap_(H_2_O_2_) was also improved by modification of all various Ni salts (nitrate, sulfate, acetate, chloride, Ni oxide, Ni hydroxide, and nickel carbonate (Table S1,† 10 wt% of Ni salt)), suggesting that the positive effect of the current density and the FE(H_2_O_2_) was caused mainly by the presence of Ni salt itself, rather than by the effect of anions. Ni nitrate showed the highest activity among them. Then, Ni nitrate was mainly used as a precursor for loading to a carbon electrode for subsequent experiments. An improved effect of the NiO-loaded KB cathode was not obvious compared to those with other nickel salts. NiO particles are hardly soluble in aqueous solution. In the case of soluble nickel-salts loaded KB cathodes, their properties were positively changed through the process of dissolution and precipitation. In the case of NiO, the bulk of NiO may be not changed in KHCO_3_ solution.

**Fig. 2:**
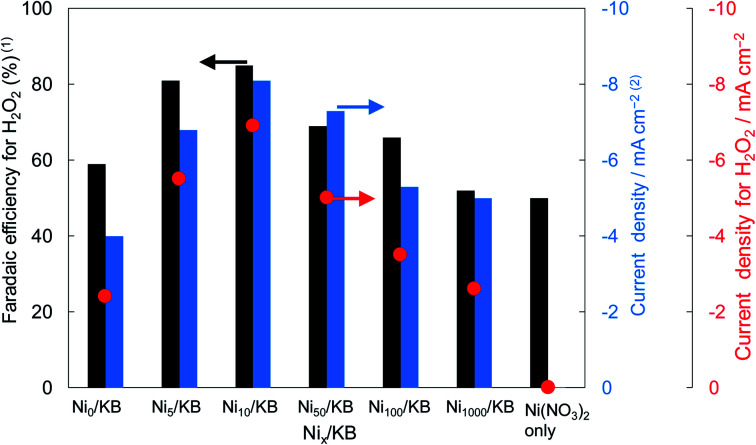
The FE(H_2_O_2_), *J*(Total), and *J*_ap_(H_2_O_2_) using various amounts of Ni nitrate on the KB cathode. The value of *x* (in weight percent) in Ni_*x*_/KB was the loading amount of Ni(NO_3_)_2_*vs.* pristine KB powder. The FE(H_2_O_2_) was measured at the passed charge after 5C. The *J*(Total) was measured at a constant potential of +0.5 V (*vs.* RHE).

**Fig. 3:**
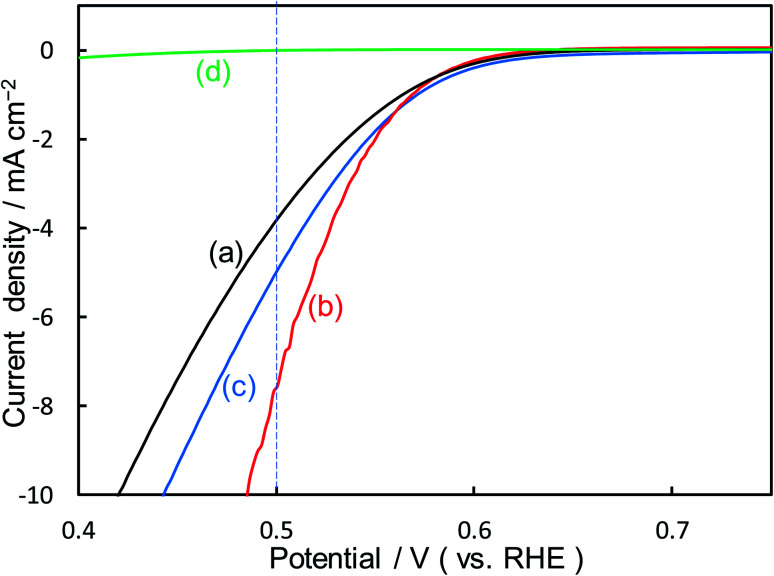
*I*–*E* curves of (a) pristine KB powder, (b) Ni_10_/KB cathode, (c) Ni_100_/KB cathode, (d) Ni nitrate only without KB powder at a scan rate of 2 mV s^−1^.

Fig. 2 shows the dependence of the loading amount of Ni nitrate over the KB cathode on FE(H_2_O_2_), *J*(Total), and *J*_ap_(H_2_O_2_). All values of the FE(H_2_O_2_), *J*(Total), and *J*_ap_(H_2_O_2_) showed volcano shape profiles, depending on the amount of Ni nitrate modification, and had the best values at 10 wt% of Ni nitrate on the KB cathode (Ni_10_/KB). As for the Ni-nitrate-loaded cathode without KB powder (the rightmost data in Fig. 2), the *J*(Total) and the *J*_ap_(H_2_O_2_) were very small, while the FE(H_2_O_2_) was equivalent to pristine KB powder. The current–potential dependences of some typical cathodes with and without Ni nitrate are shown in Fig. 3. It was surmised that the loaded nickel compound itself on the CP substrate might be hardly conductive, and the excess amount of Ni compound hindered the electron transfer though the network of the conductive KB.

**Fig. 4:**
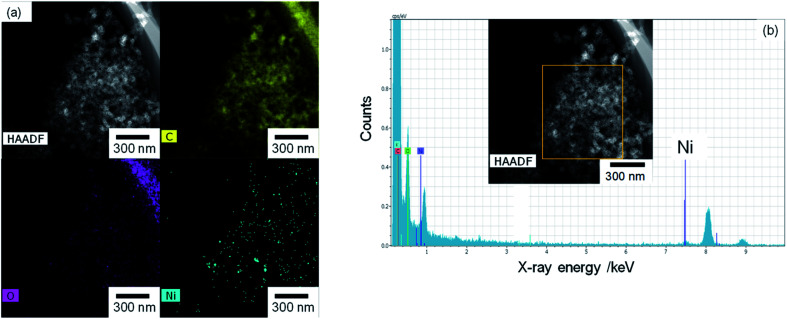
Ni_10_/KB images: (a) HAADF-TEM image and element mapping images of EDX. (b) EDX spectrum and analysis area (yellow square).

The TEM and EDX images of Ni_10_/KB are shown in Fig. 4. The Ni_10_/KB sample was prepared by immersion in KHCO_3_ electrolyte. Although it was difficult to recognize the clear particle shapes of the Ni compounds based on a TEM image only, the presence of a small aggregation of Ni element was confirmed by TEM-EDX image analysis. The aggregation size of the Ni compounds was approximately 10–40 nm in the EDX mapping images. On the other hand, when a larger amount of Ni was loaded (Ni_100_/KB), a large aggregation (>0.5 μm) of the Ni element was observed by SEM-EDX measurements (Fig. S3†). It was speculated that the large aggregation of the Ni compounds might decrease the current density at a higher loading amount of Ni(NO_3_)_2_ ≧ 50 wt% in Fig. 2.

The Ni amount with KB powder (Ni_10_/KB and Ni_100_/KB) measured by XRF before and after washing with distilled water or KHCO_3_ aqueous solutions is shown in Table 2. The Ni signal by XRF before the washing process completely disappeared after washing with distilled water. On the other hand, after washing with KHCO_3_ aqueous solution, the Ni signal was clearly detected, and more than 80% of Ni remained on the KB cathode. Ni(NO_3_)_2_ is easily dissolved in water, while nickel carbonates have a very poor water solubility (solubility product (p*K*_sp_) = 11.2).^48^ Actually, the green transparent aqueous solution of Ni(NO_3_)_2_ changed to colorless by the addition of KHCO_3_, and insoluble green powders were precipitated (Fig. S4†). The positive effect of Ni loading on the *I*–*E* curve disappeared by washing with distilled water, not by washing with a bicarbonate solution (Fig. S5†). Moreover, the presence of Ni species on the surfaces of the cathodes was also investigated by XPS (Fig. S6†). The peaks of the spectrum of Ni2p_3/2_ and Ni2p_5/2_ were observed on Ni_10_/KB (a) before and (b) after washing with KHCO_3_ aqueous solution. However, they were not observed (c) after washing with distilled water. While the apparent surface coverage of Ni compounds to carbon before washing with KHCO_3_ aqueous solution as calculated by XPS intensity was not large (less than 1%), the exact Ni coverage was difficult to estimate due to the low sensitivity and presence of carbon impurities.

**Table 2:** Amount of Ni compound on the KB cathode as measured by XRF

	Pristine KB	Ni_10_/KB	Ni_100_/KB
**Amount of Ni/μg cm** ^**−2**^			
Before washing	0	2.6	24.5
Washing with distilled water	0	0	0
Washing with 2.0 M KHCO_3_	0	2.3	20.2

**Fig. 5:**
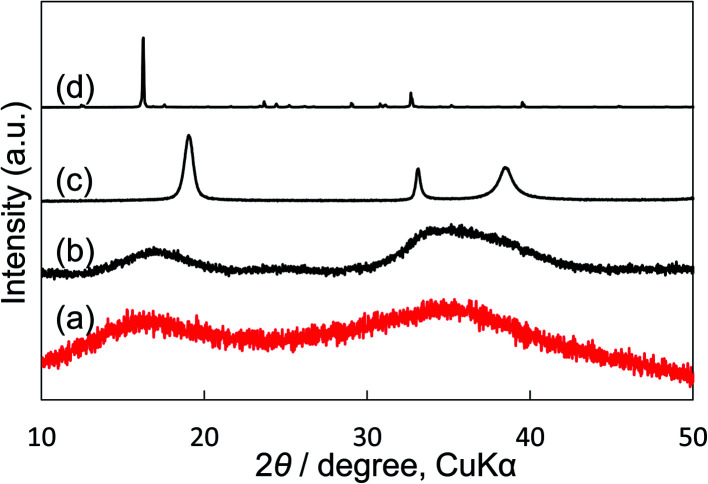
XRD patterns of (a) our synthetic sample prepared by precipitation with Ni(NO_3_)_2_ and KHCO_3_ aqueous solution. (b) Reagent powder of nickel(ii) carbonate basic hydrate (NiCO_3_·2Ni(OH)_2_·4H_2_O) as reference. (c) Reagent powder of nickel(ii) hydrate (Ni(OH)_2_) as reference. (d) Reagent powder of nickel(ii) nitrate hexahydrate (Ni(NO_3_)_2_·6H_2_O) as reference. KB powder was not mixed.

We compared our electrocatalyst powder sample with reference reagents of nickel(ii) carbonate basic hydrate (NiCO_3_·2Ni(OH)_2_·4H_2_O; Fujifilm Wako Pure Chemical Corporation, 44% as Ni), nickel(ii) hydroxide (Ni(OH)_2_; Fujifilm Wako Pure Chemical Corporation, 95%) and nickel(ii) nitrate hexahydrate (Ni(NO)_2_·6H_2_O; Fujifilm Wako Pure Chemical Corporation, 99.9%) using XRD and thermogravimetric-differential thermal analysis (TG-DTA). Our synthetic powder sample was prepared by immersing Ni(NO_3_)_2_ in a KHCO_3_ aqueous solution. As shown in the XRD patterns (Fig. 5), the shape and the broad peak position at around 17° and 35° of our powder sample were similar to those of the reference nickel carbonate rather than Ni(OH)_2_. In the TG-DTA results (Fig. S7†), the curves of TG and DTA of our electrocatalyst sample (a) were very similar to those of the reference reagent of NiCO_3_·2Ni(OH)_2_·4H_2_O (b), but different from those of Ni(OH)_2_ (c) and Ni(NO)_2_·6H_2_O (d). Therefore, it was concluded that the loaded Ni(NO_3_)_2_ on the KB cathode was immediately changed to insoluble nickel(ii) carbonate basic hydrate when the cathode electrode was soaked in a KHCO_3_ solution, and that the small particles (10–40 nm) of Ni carbonate formed by this process can function as an excellent catalyst for H_2_O_2_ production on the KB cathode. This simple preparation of the insoluble electrocatalyst at ambient conditions is a significant advantage for practical applications.

We confirmed that the H_2_O_2_ production properties on FE(H_2_O_2_) and *J*_ap_(H_2_O_2_) over Ni_10_/KB were almost the same as those over pristine KB in KOH and potassium hydrogen phosphate aqueous solution, suggesting that a positive nickel salt effect was not observed in KOH and potassium hydrogen phosphate aqueous solution, in contrast with the KHCO_3_ aqueous solution. The particle of nickel carbonate basic hydrate was stable and insoluble in KOH (pH > 14) and potassium hydrogen phosphate (pH > 8.5, which is the same as KHCO_3_) aqueous solution. The surface condition is very important for electrocatalytic reactions. The loaded Ni(OH)_2_ on KB could show a positive effect in KHCO_3_ aqueous solution, as shown in Table 1. It was confirmed using XPS measurement that the surface carbonate was reduced after the immersion in KOH or potassium hydrogen phosphate aqueous solution. From all results, it is speculated that the nickel adsorbed with carbonate, rather than nickel element itself, on the outermost surface and at the interface of the loaded electrocatalyst with KB possibly being the active site for H_2_O_2_ production.

It has been reported that the activity of a carbon-based cathode for H_2_O_2_ production from O_2_ could be improved by nitric acid treatment, where the carbon surface was changed in concentrated nitric acid for a long time at high temperature.^49,50^ Therefore, we tried to combine nitric acid treatment and Ni carbonate effect on the KB cathode (abbreviated as Ni/KB_HNO_3__). Table 3 shows the *J*(Total), FE(H_2_O_2_), and *J*_ap_(H_2_O_2_) for the O_2_ reduction reaction to H_2_O_2_ on KB_HNO_3__ and Ni_10_/KB_HNO_3__. All values of the FE(H_2_O_2_), *J*(Total), and *J*_ap_(H_2_O_2_) of KB_HNO_3__ were improved by the nitric acid treatment compared to those when the pristine KB was used. Moreover, the *J*(Total) and *J*_ap_(H_2_O_2_) of KB_HNO_3__ were improved by the loading of Ni carbonate (Ni_10_/KB_HNO_3__), and the *J*_ap_(H_2_O_2_) reached −9.8 mA cm^−2^ at +0.5 V *vs.* RHE. The *J*(Total) and *J*_ap_(H_2_O_2_) of Ni_10_/KB_HNO_3__ were higher compared to those of Ni_10_/KB, suggesting the synergistic effect of nitric acid treatment and Ni carbonate catalyst.

**Table 3:** FE(H_2_O_2_), *J*(Total), and *J*_ap_(H_2_O_2_) of nitric acid treatment and Ni loading on the KB cathodes

	FE(H_2_O_2_)^a^/%	*J*(Total)^b^/mA cm^−2^	*J*_ap_(H_2_O_2_)/mA cm^−2^
Ni_10_/KB	85	−8.1	−6.9
Ni_10_/KB_HNO_3__	82	−12.0	−9.8
KB_HNO_3__	78	−8.9	−6.9
KB	59	−4.0	−2.4

a The FE(H_2_O_2_) was measured at the passed charge after 5C.

b The *J*(Total) was measured at a constant potential of +0.5 V (*vs.* RHE).

**Fig. 6:**
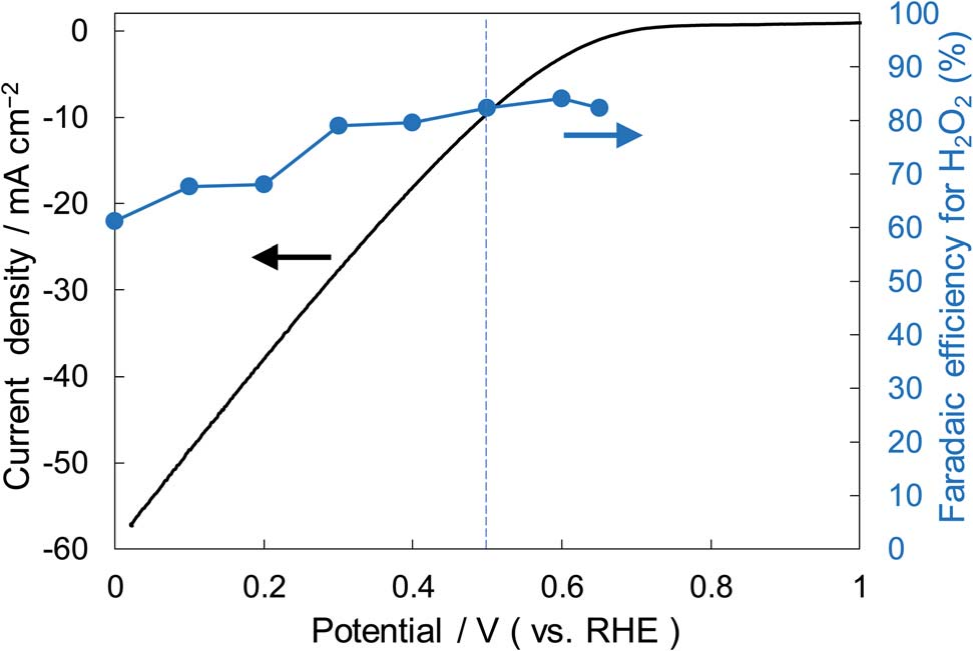
*I*–*E* curves for Ni_10_/KB_HNO_3__ (area 1 cm^2^) at a scan rate of 2 mV s^−1^ and the FE(H_2_O_2_) at the passed charge after 5C in terms of the applied potential.

**Fig. 7:**
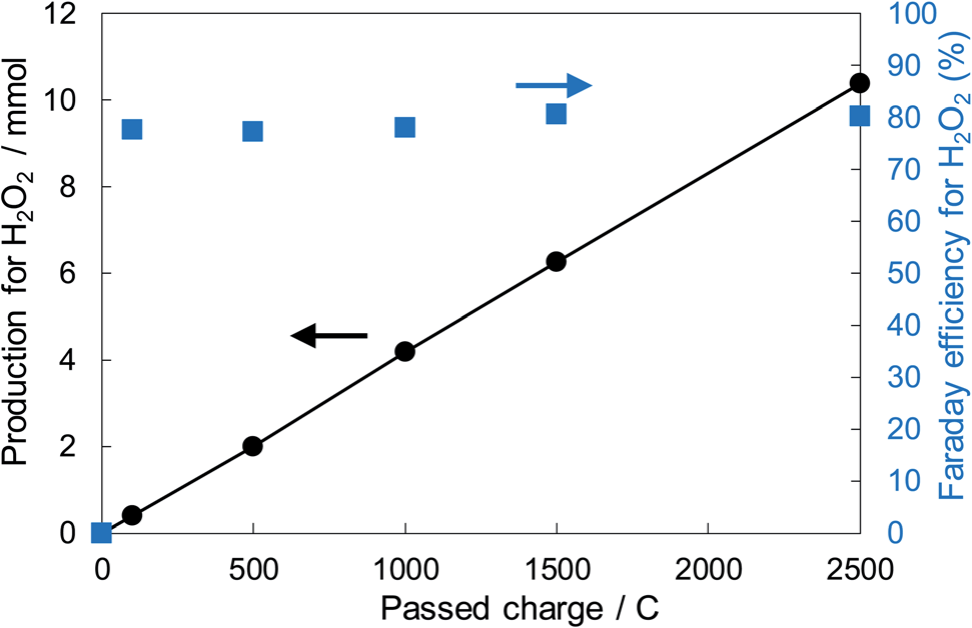
H_2_O_2_ production and the FE(H_2_O_2_) in terms of the passed charge. The cathode used was Ni_10_/KB_HNO_3__ (loading 1.5 mg cm^−2^, 20 cm^2^). The FE(H_2_O_2_) was measured at a constant potential of +0.5 V (*vs.* RHE). The volume of the electrolyte solutions of the anode and cathode chambers was 35 mL.

Fig. 6 shows the dependence of the FE(H_2_O_2_) on the applied potentials for Ni_10_/KB_HNO_3__. The FE(H_2_O_2_) increased with the applied potential positively, and this behavior was similar to those in previous reports of carbon-based cathodes in KOH.^51^ The highest FE(H_2_O_2_) at 5C was 82–84% at around +0.5 to +0.6 V (*vs.* RHE). We also measured faradaic efficiency by the rotating ring disk electrode (RRDE) method (Fig. S8†), and found that the value of the FE(H_2_O_2_) by the RRDE method was 85% at +0.5 V (*vs.* RHE), which was almost consistent with the results of the accumulated H_2_O_2_ after passing 5C electrons of using a Fe^3+^ colorimetry method. The production rates of H_2_O_2_ on Ni_10_/KB_HNO_3__ at +0.5 and +0.2 V (*vs.* RHE) were 121.9 and 330.3 mmol L^−1^ h^−1^ g^−1^ cm^−2^, respectively. To the best of our knowledge, these rates for reductive H_2_O_2_ production were the highest among all reports on noble-metal-free cathodes in aqueous solutions having near-neutral pH (Table S2†). Fig. 7 shows the long-term evaluation result of H_2_O_2_ production on the Ni_10_/KB_HNO_3__ cathode with large amounts of passed charge. H_2_O_2_ production was increased linearly at a constant applied bias at +0.5 V (*vs.* RHE), and the FE(H_2_O_2_) was around 80% passing charges up to 2500C, suggesting that the produced H_2_O_2_ was not further reduced to H_2_O sequentially (eqn (10)) for a long term. From the linear production and the agreement of the FE(H_2_O_2_) by colorimetry and RRDE methods for long- and short-term quantitative measurements, respectively, we concluded that the total H_2_O_2_ selectivity was determined by the initial H_2_O_2_ selectivity on the cathode with the electrocatalyst. The amount of the produced H_2_O_2_ was 10.4 mmol and the amount of nickel was 0.037 μmol cm^−2^ (calculated from XRF) using Ni_10_/KB_HNO_3__. Therefore, the turnover number was calculated as more than 14 000 from eqn (7). The H_2_O_2_ accumulation concentration reached 1.0 wt% in 35 mL of 2.0 M KHCO_3_ aqueous solution. In addition, the *I*–*E* curves of the cathode hardly changed even after repeated 150 cycles of *I*–*E* measurements during 36 h (Fig. S9†), and the cathodic current remained around −11 mA cm^−2^, suggesting that the modification effect of the Ni_10_/KB_HNO_3__ cathode was very stable under the H_2_O_2_ production conditions.

Nitric acid treatment and Ni carbonate catalyst had different effects. The *I*–*E* curve of the Ni_10_/KB after washing with distilled water cathode (Fig. S5(c)†) was overlapped to that of the pristine KB cathode (Fig. S5(a)†), and the FE(H_2_O_2_) of Ni_10_/KB after washing with water (62%) was almost similar as that of the pristine KB (59%). It is suggested that the Ni_10_/KB cathode returned to the original KB just by washing with pure water and reversibly due to the removal of the loaded Ni compound. On the other hand, the mechanism of the nitric acid treatment effect was explained by the change of the carbon surface structure, especially by the ratio of sp^2^ and sp^3^ bonded carbons.^51,52^ The former originated mainly from graphitic and/or aromatic bonded carbon (C=C), and the latter was from aliphatic and/or diamond bonded carbon (C–C). By XPS measurement, the sp^2^/sp^3^ ratio of C 1s became higher, and the activity was higher.^49,53^ It was also confirmed that the sp^2^/sp^3^ ratio and the peak of the COO functional group at around 289.3 eV in our samples increased after nitric acid treatment (Fig. S10†). The increase of sp^2^/sp^3^ may indicate the increase of the graphitic carbon structure with high conductivity. The C 1s XPS spectrum of KB_HNO3_ did not change even when the sample was washed with distilled water thoroughly (Fig. S11†), suggesting that the carbon surface structure of the KB powder was irreversibly oxidized or dehydrated by concentrated nitric acid treatment at 353 K.

The positive effect of nitric acid treatment was not observed when the immersed time was short (<1 h), and/or the temperature was low at around room temperature. In the case of KB_HNO3_ and Ni_10_/KB_HNO3_, the XPS spectra of C 1s were hardly changed by Ni carbonate loading (Fig. S10(c and d)†). From the results, it was concluded that the mechanism of effect by Ni carbonate catalyst was different from that by nitric acid treatment. It was surmised that the presence of small particles of insoluble Ni carbonate catalyst (mainly NiCO_3_·2Ni(OH)_2_·4H_2_O) on the carbon surface of both KB and KB_HNO3_ could accelerate the two-electron process from O_2_ to H_2_O_2_ effectively. The detailed mechanism of the nickel carbonate basic hydrate effect is under investigations.

**Fig. 8:**
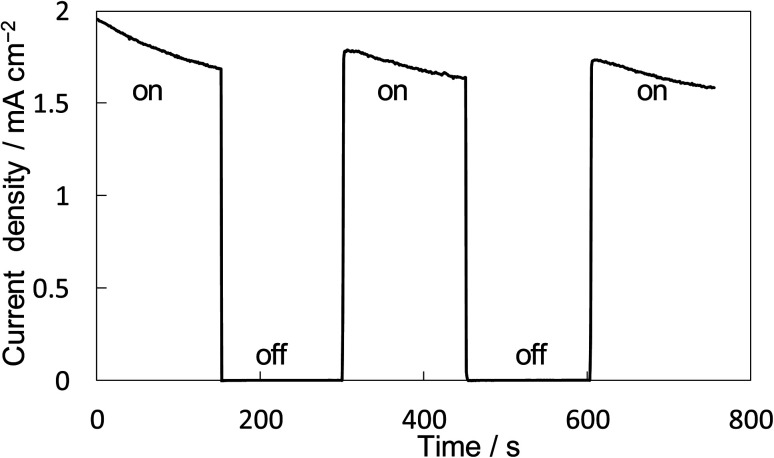
*I*–*T* curve for the BiVO_4_/WO_3_ anode and Ni_10_/KB_HNO3_ cathode without applying an external bias under illumination (AM 1.5G, 1 SUN) and dark. Measurement conditions: a two-electrode system into a one-compartment cell was used with 2.0 M KHCO_3_ aqueous solution under CO_2_ and O_2_ bubbling each for 50 mL min^−1^.

The property of H_2_O_2_ production on the BiVO_4_/WO_3_ photoanode is known to be highly excellent, and we also confirmed that the FE(H_2_O_2_) was around 90% initially on our BiVO_4_/WO_3_ photoanode in KHCO_3_ aqueous solution.^19,29,32^ Finally, the combination of the Ni_10_/KB_HNO_3__ cathode with the BiVO_4_/WO_3_ photoanode was investigated in the one-compartment cell without any membrane between electrodes in 2.0 M KHCO_3_ aqueous solution under the solar simulator AM 1.5G (1 SUN). Fig. 8 shows the current–time dependence under simulated solar light irradiation in the two-electrode system without an applied bias potential. The current was not observed between electrodes in the dark condition. When the simulated solar light was irradiated to the photoanode, a photocurrent of >1.75 mA cm^−2^ was observed initially, and the photocurrent was maintained at around 1.5 mA cm^−2^. This photocurrent value was in agreement with that in the *I*–*E* curve at 0 V of the potential (Fig. S12†). The average FE(H_2_O_2_) from both electrodes was calculated to be 168% in total at passed charge after 0.5C using eqn (5), and the production rate of H_2_O_2_ was estimated to be 0.92 μmol min^−1^ cm^−2^. The values of the apparent solar-to-chemical energy conversion efficiency for the H_2_O_2_ production (STC_H_2_O_2__) were estimated to be 1.75% without applying an external bias, using eqn (8). This value is, to the best of our knowledge, the highest among all the reported values for H_2_O_2_ production systems using simulated solar light (Table S3†). We successfully achieved highly efficient H_2_O_2_ production by only using electrolytes, oxygen, and simulated solar light, without using external electricity, a membrane, and a noble metal.

## Conclusion

We have successfully prepared a highly active non-noble-metal electrocatalyst for H_2_O_2_ production from O_2_ in 2.0 M KHCO_3_ aqueous solution using an *in situ* preparation process. The small particles (10–40 nm) of Ni carbonate prepared by immersion of Ni nitrate in a KHCO_3_ solution are remarkably effective on the HNO_3_ pretreated KB electrode. The highest values of the FE(H_2_O_2_) and *J*_ap_(H_2_O_2_) of 82% and −9.8 mA cm^−2^, respectively, were obtained using a Ni_10_/KB_HNO_3__ cathode at +0.5 V *vs.* RHE. The cathode is very stable, and the H_2_O_2_ accumulation concentration can reach 1.0 wt%. Finally, the H_2_O_2_ production on a BiVO_4_/WO_3_ photoanode and a Ni_10_/KB_HNO_3__ cathode in a one-compartment photochemical cell was demonstrated without applying an external bias, and the apparent FE(H_2_O_2_) was 168% in total. The production rate of H_2_O_2_ was 0.92 μmol min^−1^ cm^−2^. The value of STC_H_2_O_2__ was estimated to be 1.75% without applying an external bias. This H_2_O_2_ production system on both electrodes without external bias has great economic advantages compared to a system using a one-side electrode with bias. This one-compartment cell system without any membrane is very simple, and it will be developed using photocatalyst sheets as both cathode and anode electrodes for a large area application.^19^ In order to accumulate the produced H_2_O_2_ further in this system, the development of the suppression method about the H_2_O_2_ successive decomposition on the photoanode is very important.^20^ Moreover, the elucidation on the mechanism of the nickel carbonate basic hydrate at the atomic level by computational chemistry is also needed. They are under investigations.

## Conflicts of interest

There are no conflicts of interest to declare.

## Supplementary Material

RA-011-D1RA01045J-s001
