# Supplementary material for: H2O2 production on a carbon cathode loaded with a nickel carbonate catalyst and on an oxide photoanode without an external bias
Source: RSC Adv. 2021 Mar 17;11(19):11224–32. doi: 10.1039/d1ra01045j (PMC8695953; doi:10.1039/d1ra01045j)
Supplement: RA-011-D1RA01045J-s001 [file RA-011-D1RA01045J-s001.pdf]

Electronic Supplementary Material (ESI) for RSC Advances.
This journal is © The Royal Society of Chemistry 2021

Table S1 The FE(H_2O_2), $J_{(Total)}$, and J_{ap}(H_2O_2) of 10 wt% various Ni salts loaded on the KB cathodes.

Ni-salts[(1)]	FE(H_2O_2) / %[(2)]	J(Total) / mA cm^{-2} [(3)]	J_{ap}(H_2O_2) / mA cm^{-2}
$Ni(NO_3)_2$	85	−8.1	−6.9
$Ni(SO_4)$	82	−7.2	−5.9
$NiCl_2$	81	−7.6	−6.2
$Ni(COOH)_2$	82	−6.4	−5.2
NiO	72	−6.1	−4.4
$Ni(OH)_2$	79	−8.0	−6.3
$NiCO_3$	85	−8.1	−6.9
nothing	59	−4.0	−2.4

(1) The loading amount of 10 wt% Ni salt on the KB cathode was prepared by impregnation and dried at 353 K.

(2) The FE(H_2O_2) was measured at passed charge after 5 C.

(3) The $J_{(Total)}$ was measured at a constant potential of +0.5 V (vs. RHE).

Table S2 Comparison of H_2O_2 production rate, *J*(Total), and FE(H_2O_2) using noble-metal-free electrocatalyst cathode under near-neutral pH condition in electrolytes.

	pH	Potential / V (vs. RHE)	*J*(Total) / mA cm^{-2}	FE(H_2O_2) / %	H_2O_2 production rate		Refs.
					mg L^{-1} h^{-1} cm^{-2}	mmol h^{-1} g^{-1} cm^{-2}	
Ni_{10}/KB_{HNO3}	8.8	0.5	−12.0	82	177.6	121.9	this study
Ni_{10}/KB_{HNO3}	8.8	0.2	−39.0	68	481.4	330.3	this study
HPC-H24	7	0.36	−1.8	60	112.4	14.7	(A)
modified graphite felt	7	0	−2.7	81	87.9	-	(B)
polypyrrole/MWCNT	9	0.5	-	-	1.1	-	(C)
WSoyGnP	8.8	0.5	−1.1	39	20.4	31.5	(D)
carbon fiber	7	0.5	−2.1	52	0.7	6.8	(E)
Co/Carbon	7	0.2	−1.0	-	0.1	0.04	(F)
F doped porous carbon	7	−0.1	-	71	120.6	23.7	(G)

Table S3 Comparison of reported values on H_2O_2 production rates and STC(H_2O_2) using photoelectrochemical systems without applied bias.

Photoanode	Cathode	Applied bias	H_2O_2 production rate / μmol·min^{-1}·cm^{-2}	STC (H_2O_2) / %[(a)]	Reactor[(b)]	H_2O_2 production type	Refs.
$BiVO_4/WO_3$	Ni_{10}/KB_{HNO3}	No external bias	0.92	1.75	One-compartment	Both sides	This work
$BiVO_4$	Mesoporous carbon	No external bias	0.48	0.92	Two-compartment	Both sides	(M)
$Al_2O_3/BiVO_4/WO_3$	WSoyGnP	No external bias	0.02	0.04	Two-compartment	Both sides	(I)
$BiVO_4$/WO	Au	No external bias	0.08	0.15	One-compartment	Both sides	(N)
Mo-BiVO4	AQ-CNT/C	No external bias	0.27	0.51	Two-compartment	Both sides	(O)
WO_3	CoII(Ch)	No external bias	0.11	0.66	Two-compartment	Cathode side	(P)
FeO(OH)/$BiVO_4$	CoII(Ch)	No external bias	0.17	1.06	Two-compartment	Cathode side	(Q)

(1) The STC(H_2O_2) value was calculated using Eq. 8.

(2) Two-compartment reactor was used with an ion-exchange membrane.

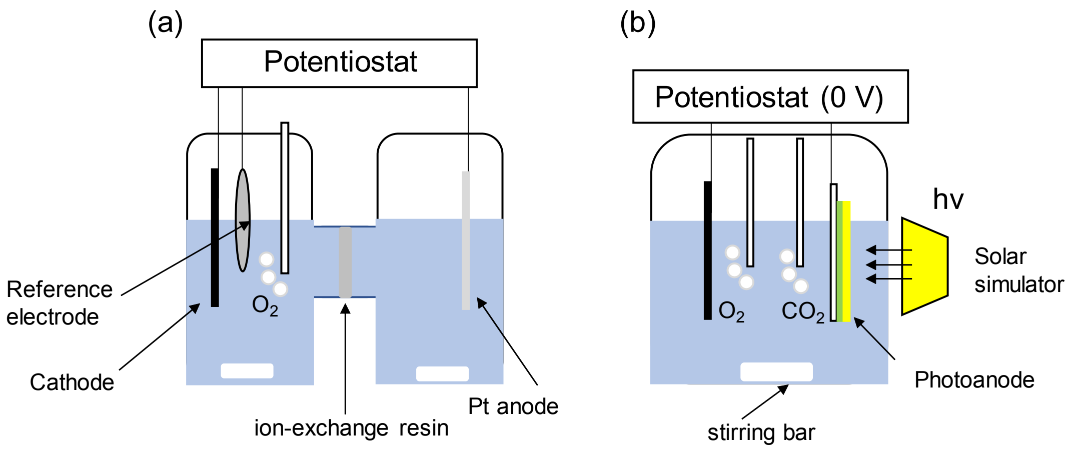

Fig. S1 Schematic illustration of electrochemical systems. (a) cathodic production of H_2O_2 through O_2 reduction in a two-compartment cell. (b) two-electrode system of photoanodic and cathodic production of H_2O_2 in a one compartment without an external bias (0 V) under solar simulated light. CO_2 and O_2 gases were bubbled each for 50 mL min^{-1} into the cell equipped with an ice bath (273–278 K) with stirring at 100 rpm.

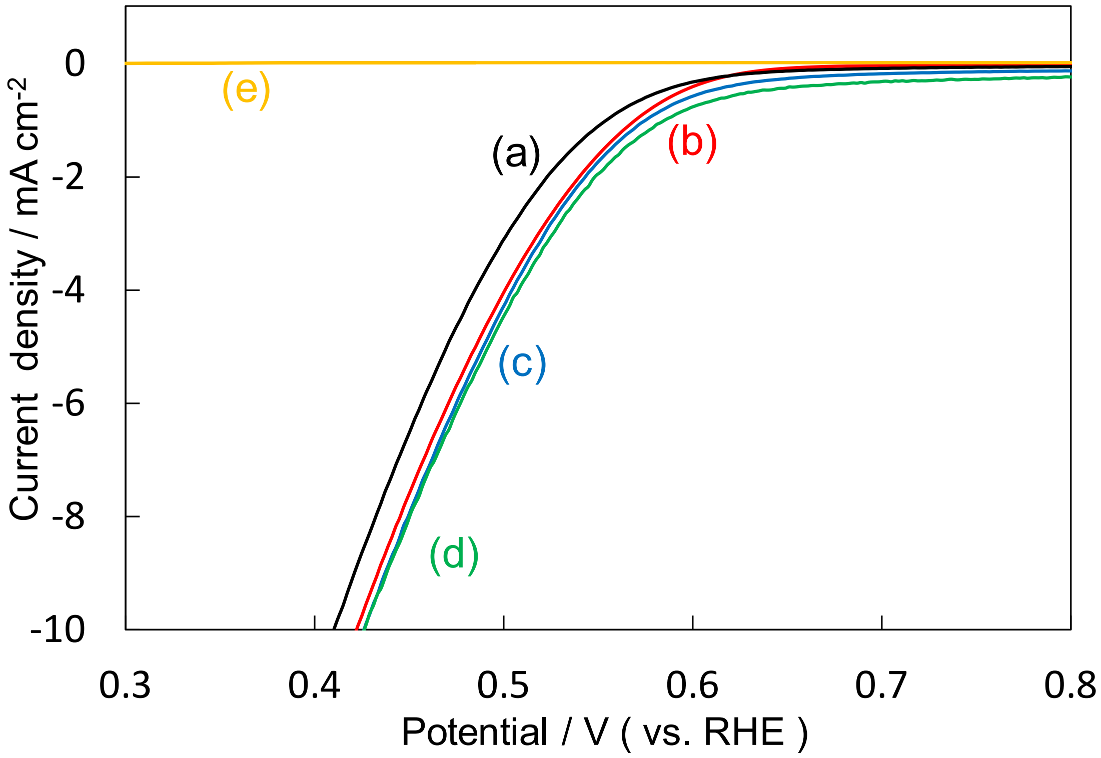

Fig. S2 *I–E* curves of the loading amount on the KB cathode: (a) 0.7 mg cm^{-2}, (b) 1.5 mg cm^{-2}, (c) 1.8 mg cm^{-2}, (d) 3.2 mg cm^{-2} at a scan rate of 2 mV s^{-1}. Exfoliation of KB powder from the CP substrate was observed in (d).

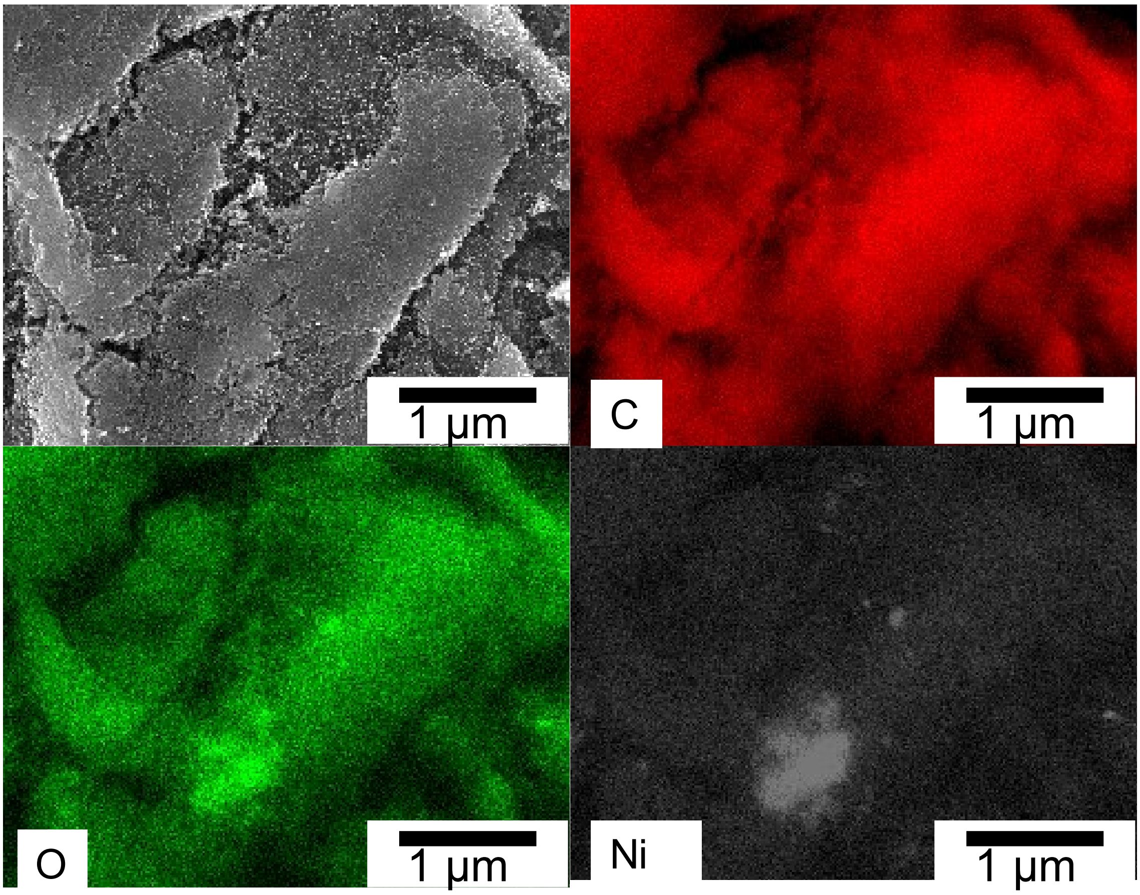

Fig. S3 SEM and EDX images of Ni_{100}/KB. Scanning electron microscope and energy-dispersive X-ray spectroscopy (SEM and EDS, Hitachi, SU9000) measurements were carried out with a field emission gun operating at 15 kV. In Ni-EDX measurement, a large aggregation (>0.5 μm) of the Ni element was observed.

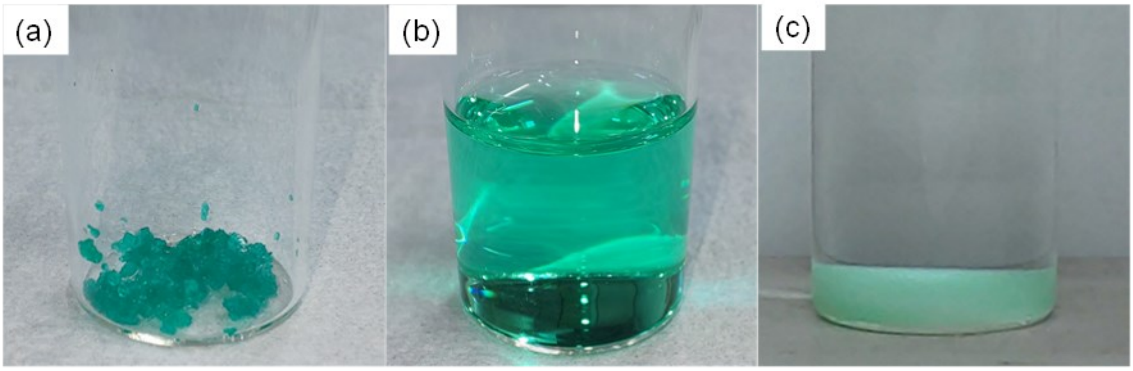

Fig. S4 (a) $Ni(NO_3)_2$ ・ $6H_2O$ (purchased from Fujifilm Wako Pure Chemicals Corporation), (b) aqueous solution of $Ni(NO_3)_2$, and (c) precipitation with 2.0 M $KHCO_3$ added to (b).

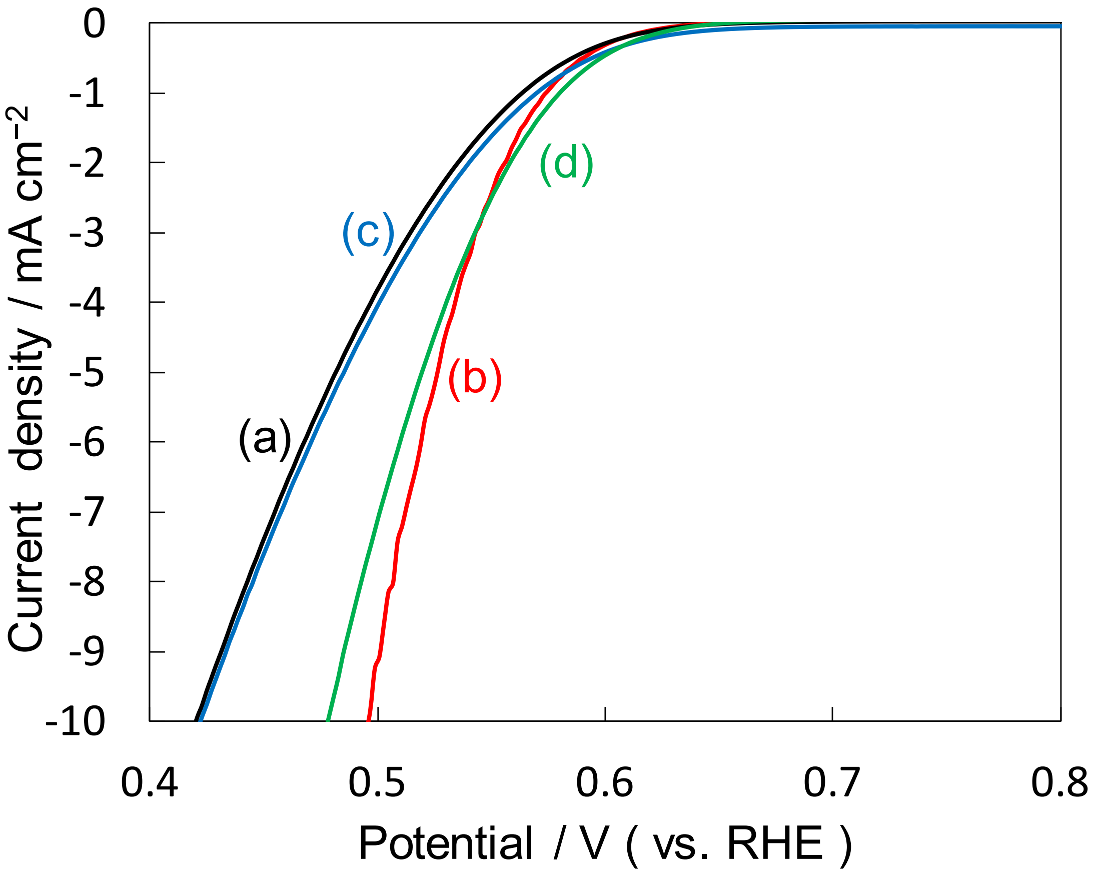

Fig. S5 *I*–*E* curves of (a) pristine KB powder, (b) Ni_{10}/KB cathode, (c) Ni_{10}/KB cathode washed with distilled water, and (d) Ni_{10}/KB cathode washed with 2.0 M $KHCO_3$ at a scan rate of 2 mV s^{-1}.

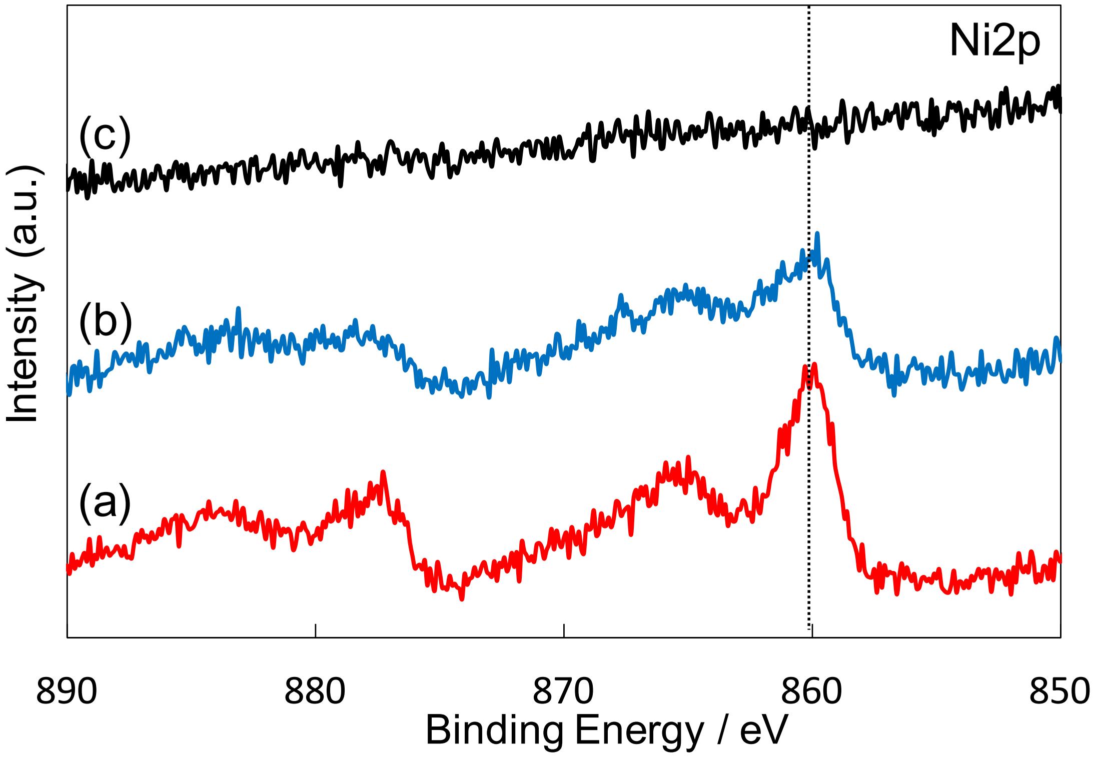

Fig. S6 Ni2*p* XPS spectra of (a) Ni_{10}/KB cathode, (b) after washing with 2.0 M $KHCO_3$ aqueous solution, and (c) after washing with distilled water.

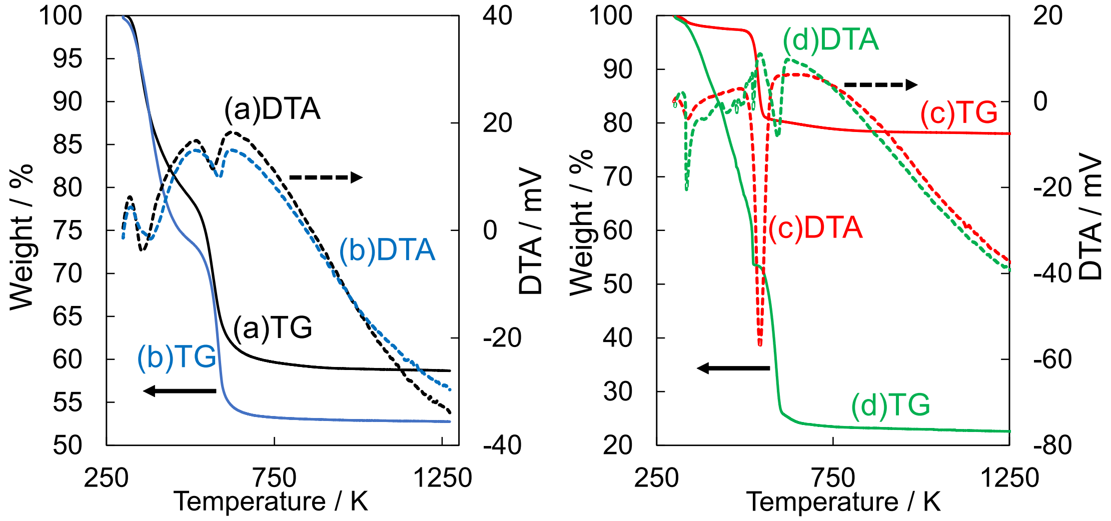

Fig. S7 Thermogravimetric–differential thermal analysis (TG–DTA) of various nickel compound powders. (a, black) powders prepared from $Ni(NO_3)_2$ precipitated by a $KHCO_3$ aqueous solution as in the preparation conditions of Ni/KB. (b, blue) purchased $NiCO_3 \cdot 2Ni(OH)_2 \cdot 4H_2O$ powders as a reference sample. (c, red) purchased $Ni(OH)_2$ powders as a reference sample. (d, green) purchased $Ni(NO_3)_2 \cdot 6H_2O$ powders as a reference sample.

TG-DTA (Rigaku, TG8120) was carried under air. The temperature range is from ambient to 1273 K with a heating rate from 5 K min^{-1}.

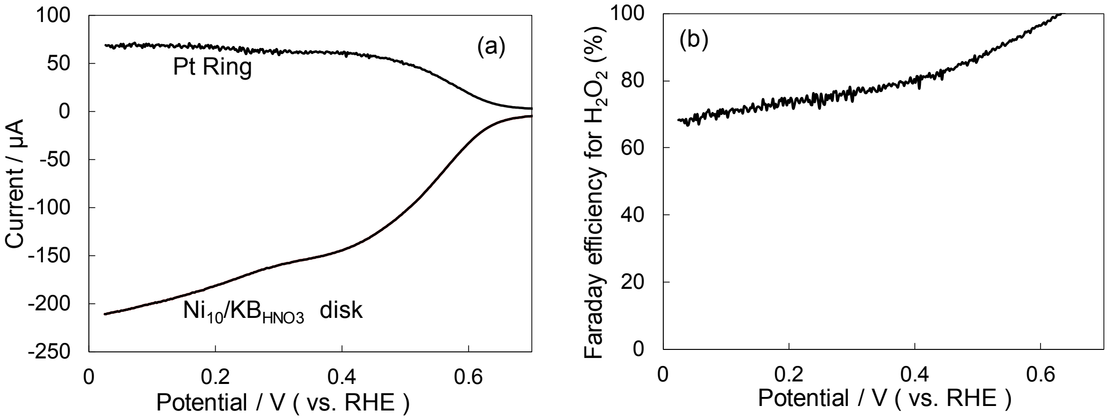

Fig. S8 Linear sweep voltammetry of a rotating ring disk electrode (RRDE) with (a) the ring current collected on a Pt ring at a constant potential of +0.1 V (vs. RHE) and the Ni_{10}/KB_{HNO3} loaded on the disk geometric current. (b) Faradaic efficiency for H_2O_2. Measurements were performed in O_2-saturated 2.0 M $KHCO_3$ at a scan rate of 2 mV s^{-1} at 1,000 rpm at room temperature.

Electrochemical experiments were conducted using an electrochemical analyzer (Hokuto Denko, HZ7000) equipped with a rotating ring disk electrode system (BAS Instruments, RRDE-3A). A standard three-electrode cell containing a counterelectrode (Pt wire), a reference electrode (Ag/AgCl), and a rotating ring disk electrode (working electrode, from BAS Instruments) was used. The working electrodes were constructed using the configuration proposed by Paulus et al.(A) and Assumpc¸ão et al.(B-D) and supported on a central glassy carbon (area = 0.1256 cm^2) and a Pt ring (area = 0.1886 cm^2). $KHCO_3$ aqueous solution (2.0 M) was used as the electrolyte. The electrode rotation rate was varied from 1,000 rpm. During the ORR measurements, the electrolyte was first saturated with oxygen for 30 min, and the flow was maintained over the electrolyte during the experiment. The measurements were performed at a fixed scan rate of 2 mV s^{-1} at room temperature. Faradaic efficiency for H_2O_2 production (FE(H_2O_2)) was calculated according to Eq. 1:

$$FE(H_2O_2)(\%) = 200 \times I_{ring} / (N \times -I_{disk} + I_{ring}) \quad (1)$$

where I_{ring} is the ring current and I_{disk} is the disk current. (E)

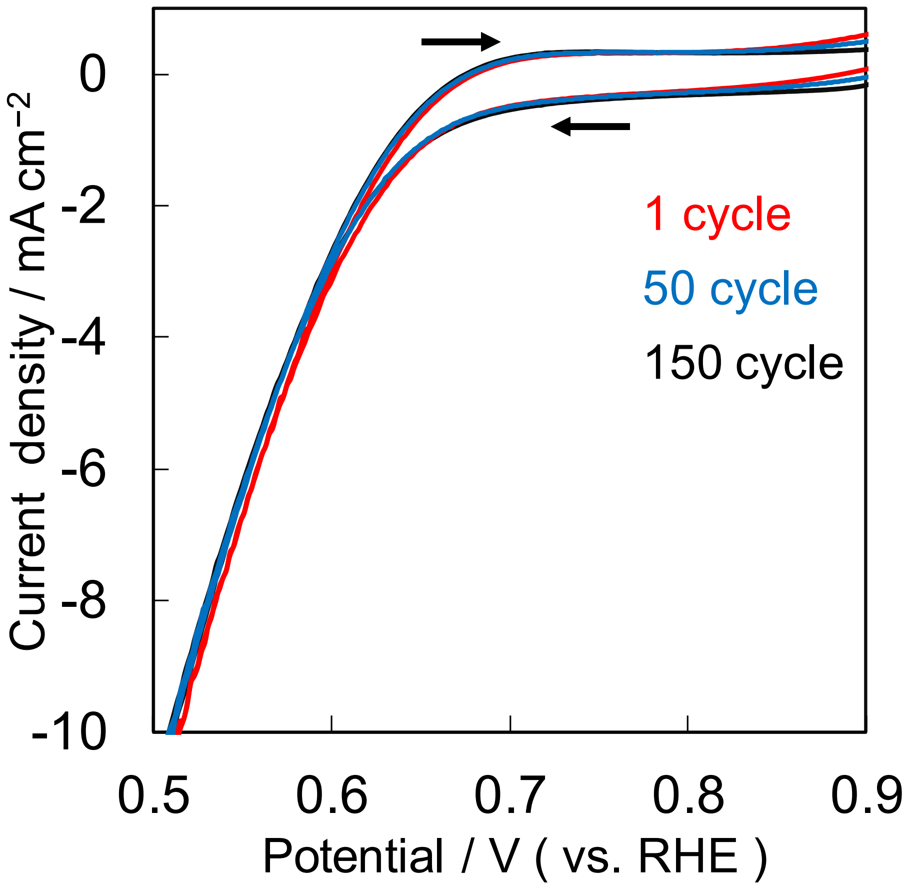

Fig. S9 Multiple-cycle tests on *I*–*E* curves of Ni_{10}/KB_{HNO3} before and after potential sweeps (+0.9 to +0.5 V vs. RHE) at a scan rate of 2 mV s^{-1}.

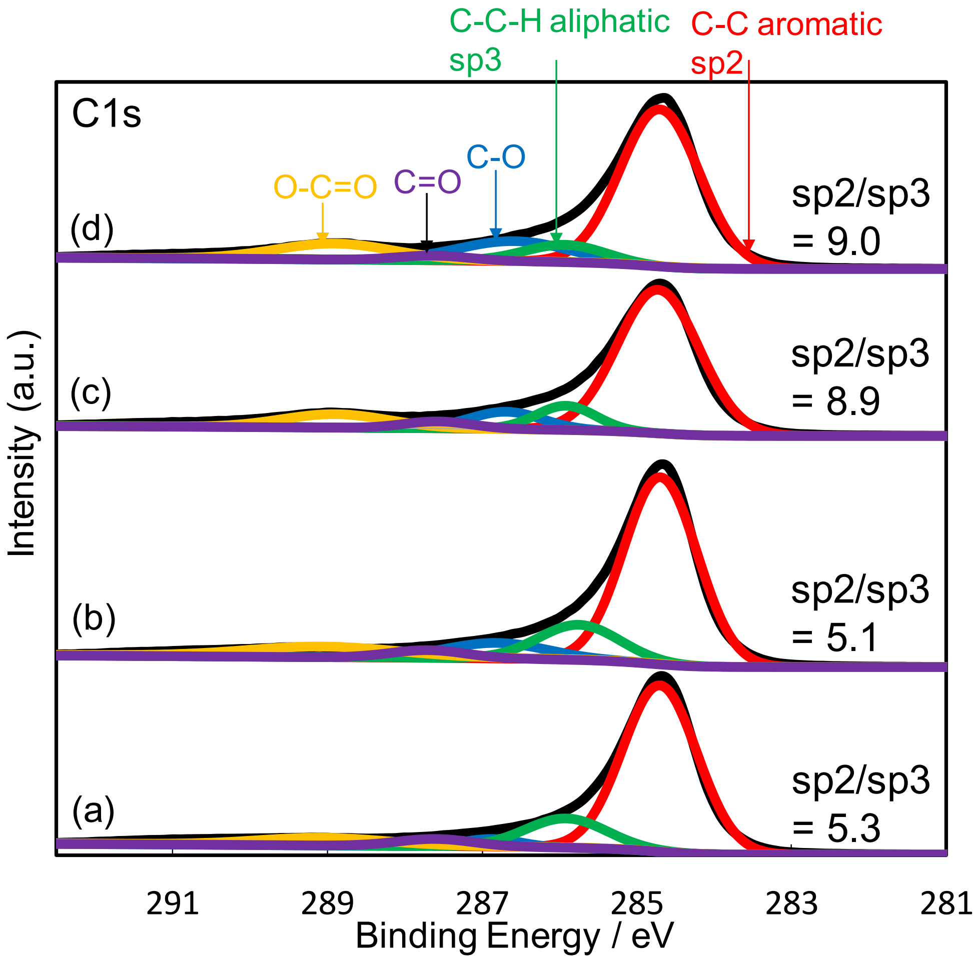

Fig. S10 C1*s* XPS spectra of (a) pristine KB powder, (b) Ni_{10}/KB cathode, (c) KB_{HNO3} cathode, and (d) Ni_{10}/KB_{HNO3} cathode

X-ray photoelectron spectroscopy (XPS, Ulvac-Phi, XPS1800) was carried out using an Al-Kα source (hv = 1,486.6 eV) with a neutralizing gun, and the vacuum in the analysis chamber was maintained at 10^{-8} atm. C1*s* and Ni2*p* spectra were acquired with a 0.1 eV step^{-1} an energy interval.

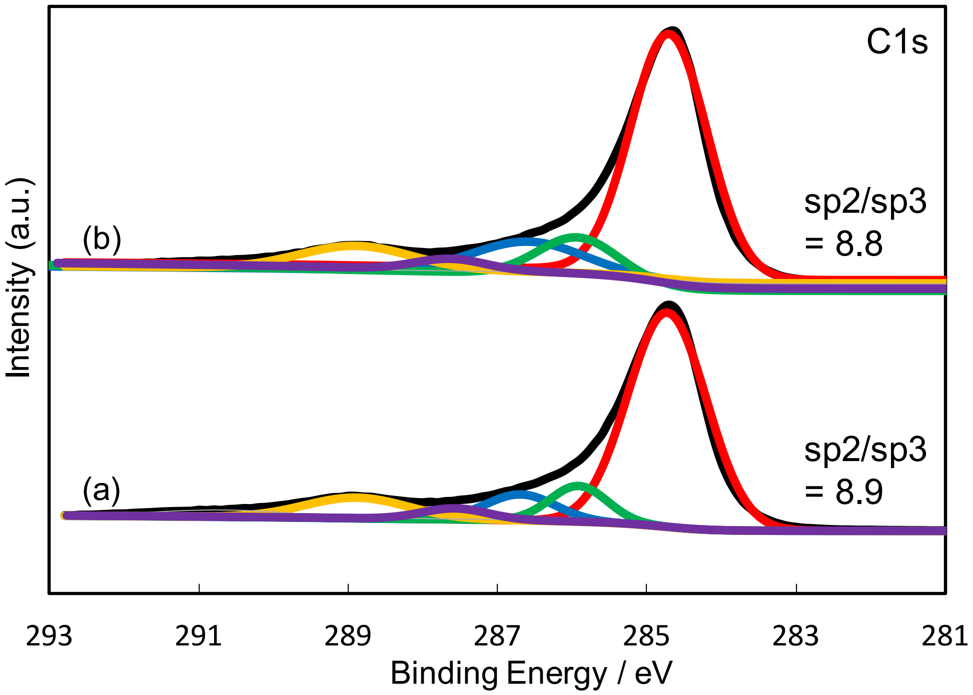

Fig. S11 C1*s* XPS spectra of (a) KB_{HNO3} and (b) after washing with distilled water.

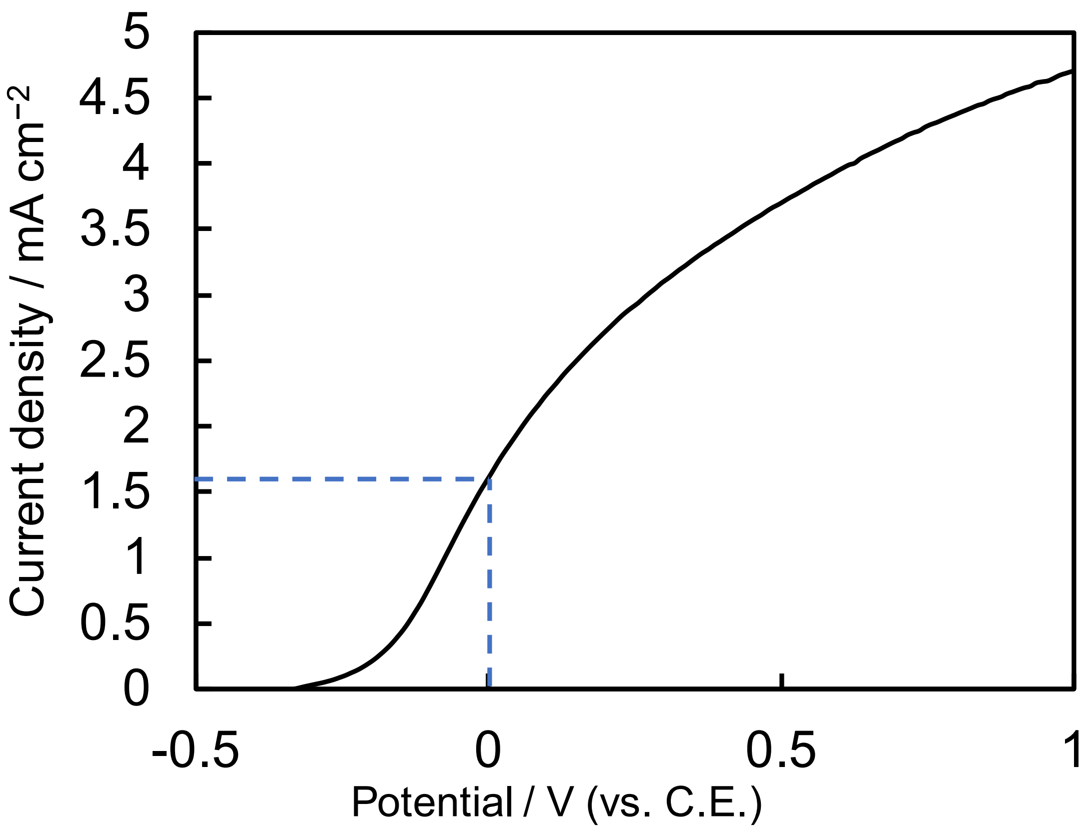

Fig. S12 *I–E* curve using $BiVO_4/WO_3$ anode and Ni_{10}/KB_{HNO3} cathode. Measurement conditions are as follows: a two-electrode system in a one-compartment cell was used with 2.0 M $KHCO_3$ aqueous solution under CO_2 and O_2 bubbling each for 50 mL min^{-1} at a scan rate of 10 mV s^{-1} under illumination (AM1.5G, 1SUN).

References

(A) U.A. Paulus, T.J. Schmidt, H.A. Gasteiger, R.J. Behm, J. Electroanal. Chem. **495**(2001) 134–145.

(B) M.H.M.T Assumpc¸ ão, A. Moraes, R.F.B De Souza, I. Gaubeur, R.T.S. Oliveira, V.S.Antonin, G.R.P. Malpass, R.S. Rocha, M.L. Calegaro, M.R.V. Lanza, M.C. Santos,Appl. Catal. A: Gen. **411-412** (2012), 1-6.

(C) M.H.M.T. Assumpc¸ ão, R.F.B. De Souza, D.C. Rascio, J.C.M. Silva, M.L. Calegaro,I. Gaubeur, T.R.L.C. Paixão, P. Hammer, M.R.V. Lanza, M.C. Santos, Carbon **49** (2011) 2842–2851.

(D) M.H.M.T. Assumpcao, D.C. Rascio, J.P.B. Ladeia, R.F.B. De Souza, E.T. Neto, M.L.Calegaro, R.T.S. Oliveira, I. Gaubeur, M.R.V. Lanza, M.C. Santos, Int. J. Elec-trochem. Sci. **6** (2011) 1586–1596.

(E) C. H. Choi, H. C. Kwon, S. Yook, H. Shin, H. Kim, and M. Choi, *J. Phys. Chem. C* 2014, **118**, 30063−30070.

(F) Y. Liu, X. Quan, X. Fan, H. Wang and S. Chen, *Angew. Chem. Int. Ed.*, 2015, **54**, 6837-6841.

(G) L. Zhou, M. Zhou, Z. Hu, Z. Bi and K. G. Serrano, *Electrochim. Acta,* 2014, **140**, 376-383.

(H) R. Babaei-Sati and J. B. Parsa, *J. Ind. Eng, Chem.*, 2017, **52**, 270-276.

(I) Y. Miyase, S. Takasugi, S.Iguchi, Y. Miseki, T. Funaki, T. Gunji, K. Sasaki, E. Fujita and K. Sayama, *Sustain. Energy Fuels*, 2018, **2**, 1621-1629.

(J) J. Choi, S.H. Hwang, J. Jang and J. Yoon, *Electrochem. Commun.*, 2013, **30**, 95-98.

(K) A. Bonakdarpour, D. Esau, H. Cheng, A. Wang, E. Gyenge and D. P. Wilkinson, *Electrochim. Acta*, 2011, **56**, 907-9081.

(L) K. Zhao, Y. Su, X. Quan, Y. Liu, S. Chen and H. Yu, *J. Cataly.*, 2018, **357**, 118-126.

(M) X. Shi, Y. Zhang, S. Siahrostami and X. L. Zheng, *Adv. Energy Mater.*, 2018, **8**, 1801158.

(N) K. Fuku, Y. Miyase, Y. Miseki, T. Funaki, T. Gunji and K. Sayama, *Chem. Asian J.*, 2017, **12**, 1111-1119.

(O) T. H. Jeon, H. Kim, H. Kim and W. Choi, *Energy Environ. Sci.*, 2020, **13**, 1730-1742.

(P) K. Mase, M. Yoneda, Y. Yamada and S. Fukuzumi, *Nat. Commun.*, 2016, **7**, 11470.

(Q) K. Mase, M. Yoneda, Y. Yamada and S. Fukuzumi, *ACS Energy Lett.*, 2016, **1**, 913-919.
